# Next Generation Sequencing of 134 Children with Autism Spectrum Disorder and Regression

**DOI:** 10.3390/genes11080853

**Published:** 2020-07-25

**Authors:** Jiani Yin, Chun-An Chun, Nikolay N. Zavadenko, Natalia L. Pechatnikova, Oxana Yu. Naumova, Harsha V. Doddapaneni, Jianhong Hu, Donna M. Muzny, Christian P. Schaaf, Elena L. Grigorenko

**Affiliations:** 1 Molecular and Human Genetics Department, Baylor College of Medicine, Houston, TX 77030, USA; yinjiani900129@gmail.com (J.Y.); chunanc@bcm.edu (C.-A.C.); schaaf@bcm.edu (C.P.S.); 2 Jan and Dan Duncan Neurological Research Institute, Texas Children’s Hospital, Houston, TX 77030, USA; 3 Department of Neurology, Center for Autism Research and Treatment, Semel Institute, David Geffen School of Medicine, University of California, Los Angeles, CA 90095, USA; 4 Neurology, Neurosurgery and Medical Genetics, Department of Pediatrics, Pirogov Russian National Research Medical University, 117997 Moscow, Russia; zavadenko@mail.ru (N.N.Z.); Funny85@bk.ru (N.L.P.); 5 Texas Institute for Measurement, Evaluation, and Statistics, University of Houston, Houston, TX 77024, USA; oksana.yu.naumova@gmail.com; 6 Vavilov Institute of General Genetics, Russian Academy of Sciences, 119991 Moscow, Russia; 7 Laboratory of Translational Developmental Sciences, Saint Petersburg State University, 199034 Saint Petersburg, Russia; 8 Human Genome Sequencing Center, Baylor College of Medicine, Houston, TX 77030, USA; doddapan@bcm.edu (H.V.D.); Jianhong.Hu@bcm.edu (J.H.); donnam@bcm.edu (D.M.M.); 9 Institute of Human Genetics, Heidelberg University, 69120 Heidelberg, Germany

**Keywords:** autism, developmental regression, exon capture and sequencing, variant classification, ACMG standards and guidelines

## Abstract

Approximately 30% of individuals with autism spectrum disorder (ASD) experience developmental regression, the etiology of which remains largely unknown. We performed a complete literature search and identified 47 genes that had been implicated in such cases. We sequenced these genes in a preselected cohort of 134 individuals with regressive autism. In total, 16 variants in 12 genes with evidence supportive of pathogenicity were identified. They were classified as variants of uncertain significance based on ACMG standards and guidelines. Among these were recurring variants in *GRIN2A* and *PLXNB2*, variants in genes that were linked to syndromic forms of ASD (*GRIN2A*, *MECP2*, *CDKL5*, *SCN1A,* *PCDH19*, *UBE3A*, and *SLC9A6*), and variants in the form of oligogenic heterozygosity (*EHMT1*, *SLC9A6*, and *MFSD8*).

## 1. Introduction

About thirty percent of individuals with ASD experience developmental regression [1]. These individuals commonly manifest cognitive decline, loss of language skills, seizures, and difficulty in managing behavior [1]. Symptoms can manifest as early as infancy or early childhood. ASD and developmental regression are involved in a few neurological conditions with known etiologies, such as Rett syndrome, Landau–Kleffner syndrome, or Christianson syndrome [1,2]. In addition, genetic variants in isolated cases of autism and developmental regression have been reported. For example, an R426H homozygous variant in the *ADSL* gene was identified in three affected individuals of the same family [3]. A translocation t (14;21)(q21.1; p11.2), disrupting the expression of *LRFN5*, was reported in a severely autistic girl who manifested regression of skills after 2 years old [4]. A female who manifested neuronal ceroid lipofuscinosis with Rett-like onset was found to bear compound heterozygous variants of *MFSD8* and a heterozygous variant in *CLN5* [5]. A boy with autism and two episodes of neurodevelopmental regression was reported to have a frameshifting variant in *TMLHE* [6]. However, beyond these aforementioned cases, the genetic and pathophysiologic underpinnings of developmental regression remain largely unknown, and it remains challenging to provide a prognosis for individuals with regressive autism.

Next generation sequencing has proven to be a powerful way to identify variants in many neurological disorders [7,8,9,10,11,12,13]. To explore the genetic predisposition to a regressive form of autism, we performed targeted exon capturing and the sequencing of candidate genes. We recruited a cohort of 141 individuals diagnosed with ASD and developmental regression and screened them for variants in genes that had previously been reported in the manifestation of these traits.

## 2. Materials and Methods

### 2.1. Patient Recruitment

The criteria for recruitment included a diagnosis of ASD, a history of severe developmental regression, and no known cause of either at the time of recruitment. Developmental regression was defined by a loss of acquired functions, as documented by a pediatrician or child neurologist or psychiatrist in the child’s medical records and a referral for a consultation for developmental regression to a specialized central medical center in Moscow, Russia. Altogether, 141 patients were recruited from 2011 to 2013. All experiments were conducted in accordance with the IRB’s requirements from St. Petersburg State University and Yale University.

### 2.2. Literature Search for Candidate Genes

Given the limited number of variants reported in cases with regressive autism, we searched for variants that were associated with developmental regression both with and without autism. We searched for the terms “autism AND regression” or “developmental regression” in the PubMed database. Then, we read the abstracts and results to confirm the presence of autism and developmental regression in the reported cases. In these cases, gene names were noted if variants were found or if the genes were involved in a copy number variation. A total of 1659 abstracts were reviewed by August 2015, which resulted in a list of 47 genes (Table 1) [2,3,4,5,6,14,15,16,17,18,19,20,21,22,23,24,25].

### 2.3. Library Preparation

Genomic DNA was isolated from whole frozen EDTA-blood (1–2 mL) using the phenol chloroform extraction method [26,27]. DNA samples were constructed into Illumina paired-end pre-capture libraries using Beckman robotic workstations (Biomek FX and FXp models). In total, 250 ng of DNA per sample was used as input for the library preparation. 

Whole Genome Amplification was completed on 47 samples, the total DNA amount of which was less than 250 ng. Briefly, DNA in a 70 μL volume was sheared into fragments of approximately 500–700 base pairs in a Covaris plate with a E220 system (Covaris, Inc., Woburn, MA, USA). The fragmented DNA was purified using 0.8× AMPure XP (Beckman, Cat. No. A63882) followed by end-repair (NEBNext End-Repair Module; Cat. No. E6050L), A-tailing (NEBNext^®^ dA-Tailing Module; Cat. No. E6053L), and ligation of the Illumina multiplexing PE adaptors with 96 different 9-bp barcode sequences using ExpressLink™ T4 DNA Ligase (a custom product from LifeTech). Pre-capture Ligation Mediated-PCR (LM-PCR) was performed for 6–8 cycles using the Library Amplification Readymix containing KAPA HiFi DNA Polymerase (Kapa Biosystems, Inc., Cat # KK2612). Universal primers, IMUX-P1.0 and IMUX-P3.0, were used in PCR amplification. AMPure XP beads (0.8×) were used to purify reaction contents after each enzymatic reaction. Libraries were quantified and their sizes were estimated using a Fragment Analyzer capillary electrophoresis system (Advanced Analytical Technologies, Inc., Ames, IA, USA). The complete protocol and oligonucleotide sequences are accessible from the HGSC website (https://www.hgsc.bcm.edu/sites/default/files/documents/Protocol-Illumina_Whole_Exome_Sequencing_Library_Preparation-KAPA_Version_BCM-HGSC_RD_03-20-2014.pdf).

Seven samples failed to pass quality control; correspondingly, we proceeded with 134 samples.

### 2.4. Sequence Capture and Sequencing on HiSeq 2000

For capture enrichment, pools of libraries (~104 ng/library and 48 libraries/pool) were prepared, and 5.0 ug/pool was used for in-solution hybridization to custom capture a design [150825_HG19_10bp_Mende_chr_EZ_HX3] that covered all exons and 10 bp intron-exon boundary regions on each side of the exons of the 47 genes (Nimblegen).

Human COT1 DNA and full-length blocking oligonucleotides (Sigma-Aldrich Inc., St. Louis, MO, USA) were added into the hybridization to block repetitive genomic sequences and the adaptor sequences. Hybridization was done at 56 °C O/N. Post-capture LM-PCR amplification was performed using the Library Amplification Readymix containing KAPA HiFi DNA Polymerase (Kapa Biosystems, Inc., Cat # KK2612) with 12 cycles of amplification. After the final AMPure XP bead purification, quantity and size of the capture library was analyzed using an Agilent Bioanalyzer 2100 DNA Chip 7500. The enriched library pools (48 libraries/pool or 96 per lane) were sequenced on a HiSeq 2000 to generate 2 × 100 bp reads. On average, for these 134 samples, including 47 samples that were whole genome amplified, 397 Mb of mapped sequence data (295 Mb unique with 537.38× sequence coverage) across the length of the targeted region were generated. On average, 96% of bases in the design were covered at a ≥ 20× depth.

### 2.5. Primary Data Analysis

The initial sequence analysis was performed using the HGSC Mercury analysis pipeline [28,29]. In summary, the bcl files produced on-instrument were first transferred into the HGSC analysis infrastructure by the HiSeq Real-time Analysis module and Mercury; we then ran the vendor’s primary analysis software (CASAVA) to de-multiplex the pooled samples and generate sequence reads and base-call confidence values (qualities). Reads were then mapped to the GRCh37 Human reference genome (http://www.ncbi.nlm.nih.gov/projects/genome/assembly/grc/human/) using the Burrows-Wheeler aligner (BWA, http://bio-bwa.sourceforge.net/). In this step, the resulting BAM (binary alignment/map) files underwent quality recalibration using GATK (http://www.broadinstitute.org/gatk/) BAM sorting, duplicate read marking, and realignment to improve indel discovery. BAM files were used by the ATLAS 2 suite (https://www.hgsc.bcm.edu/software/atlas-2) to call single nucleotide variants (SNV) and insertion/deletion variants and produce a variant call file (VCF). Finally, annotation data were added to the VCF using a suite of annotation tools (Cassandra) that brings together frequency, function, and other relevant information, using AnnoVar with UCSC and RefSeq gene models, as well as a host of other internal and external data resources (https://www.hgsc.bcm.edu/software/cassandra).

### 2.6. Sanger Sequencing

Mutation validation was performed with bidirectional sequencing of the selected sample sites. PCR reactions were prepared using 5 ng of genomic DNA, 0.4 µM oligonucleotide primers, and 0.7× Qiagen Multiplex Master Mix (Cat. no. 206145) containing HotStar Taq, buffer, and polymerase. Two reactions were completed for each sample site, one with and one without, using the QSOL PCR additive provided in the kit. Touchdown PCR was performed with the following parameters: 98 °C for 5 min and 10 cycles of 98 °C for 30 s, 72 °C for 30 s (decreasing by 1 °C per cycle), and 72 °C for 1 min. The reaction then continued with 30 cycles of 98 °C for 30 s, 63 °C for 30 s, and 72 °C for 1 min, followed by a final extension at 72 °C for 5 min. The PCR products were purified with a 1:15 dilution of Exo-SAP, diluted by 0.6×, and then cycle-sequenced for 25 cycles using a 1/32nd dilution of a BigDye Terminator v3.1 reaction mix (Applied Biosystems, Cat. No. 4337456). Finally, the reactions were precipitated with ethanol, resuspended in 0.1 mM EDTA, and analyzed on AB 3730xl sequencing instruments using the Rapid36 run module and 3xx base-caller. SNPs were identified and visually validated with a Mutation Surveyor.

## 3. Results

### 3.1. Targeted Sequencing of Regressive Autism Candidate Genes

We sequenced 47 candidate genes in 134 individuals with autism and severe developmental regression using targeted capture and sequencing. Candidate genes were selected based on at least one case of developmental regression, with or without autism, reported in the literature prior to August 2015, with either a single nucleotide variant in the gene or copy number variation involving the gene (Methods section “Literature search for candidate genes”). A custom library was designed to capture all exons and intron-exon boundary regions of the 47 genes. 

Initial variant calling yielded 2121 variants. We filtered these variants based on the following criteria: (1) Nonsense, missense, frameshift, or splice-region variant type (Synonymous, inframe, and variants in the UTR region were excluded); (2) a minor allele frequency lower than 2 × 10^−4^, or 1 in 5000 individuals, in the ExAC database or 1000 Genomes; (3) prediction to be damaging or possibly damaging by SIFT and/or PolyPhen; and (4) 10 or fewer instances of calls among these 134 individuals (variants called more than 10 times were considered likely false positives). After filtering, 130 variants remained to be validated. Of those, we were able to confirm 41 of the filtered variants using Sanger sequencing, among which 16 unique variants had evidence supportive of pathogenicity based on the ACMG criteria. All 16 variants were classified as “uncertain significance” based on the ACMG standards and guidelines for the interpretation of sequence variants [30]. Among the remaining 89 variants, 65 were false positives, and another 34 were not confirmed due to insufficient DNA amount or failure of PCR amplification of the variant region. The clinical and genetic data of the 15 individuals carrying variants of uncertain significance, as well as evidence supportive of pathogenicity of the variants, are summarized in Table 2.

### 3.2. Recurrent Variants Identified in Our Regressive Autism Cohort

In our sequenced cohort of 134 individuals with autism and regression, we identified two recurrent variants, *GRIN2A* c.28C > A (p.Leu10Met) and *PLXNB2* c.742C > T (p.Arg248Cys). The variant in *GRIN2A* was novel and was predicted to be damaging using both SIFT and PolyPhen. Heterozygous *GRIN2A* germline variants are considered to be major genetic causes of Landau Kleffner syndrome, which is characterized by speech impairment and focal epilepsy [31]. Two unrelated males in our cohort were found to harbor the variant *GRIN2A* c.28C > A. Both were referred for evaluation at 3 years of age and had language development arrest and regression. In addition, one of the males (ID113) had hydrocephaly and showed an increase in emotional negativity and stereotypies. The other male (ID090) experienced sleep disturbance.

The *PLXNB2* gene is important for brain development [32,33,34] but has not been associated with any disorder. The deletion of *PLXNB2* and other genes was reported in an individual with autism and speech impairment [35]. Unrelated individuals ID086 (male) and ID110 (female) had seizures and regression and were found to harbor variants in *PLXNB2*. The male was referred at the age of 14 years; retrospectively, he experienced the onset of febrile seizures at the age of 3 years, which intensified and occurred regularly 2–3 times a night. The girl was referred at the age of 10 years; retrospectively, febrile seizures were reported with onset at the age of 1 year. In both children, the progression of seizures was coupled with the regression of language and cognitive functions. Recently, the female was diagnosed with Angelman Syndrome using the PRC test based on the allelic methylation differences at the *SNRPN* locus (15q11.2). In both cases, *PLXNB2* c.742C > T was the only candidate variant from our sequencing analysis. This variant was found in the ExAC database at a rate of 4.1 × 10^−5^ and was predicted to be damaging using SIFT, PolyPhen, and MutationTaster. Due to the lack of a proper control cohort, we were not able to assess the frequency of this variant in the normal population of the same ethnicity.

### 3.3. Variants in Genes Associated with Syndromic Forms of ASD

Rett syndrome, caused by mutations in *MECP2*, is one well-established cause of developmental regression and autism, which accounts for roughly 0.5% of such cases. We identified a missense variant c.403A > G (p.Lys147Glu) in *MECP2* while sequencing one female (ID073), regardless of excluding individuals known to carry pathogenic variants in *MECP2*. This girl was referred at the age of 7 years. Her developmental history indicated the presence of microcephaly and the onset of seizures at the age of 6 months, followed by arrested development and a rapid loss of acquired skills. This variant in *MECP2* was absent from the ExAC and gnomAD databases, was predicted to be damaging, and was reported as pathogenic or with uncertain significance in two separate records (ClinVar#143564). We believe that autism and the developmental regression of ID073 are likely attributed to this variant. 

Genetic variants in *CDKL5* cause a disorder that is characterized by early onset seizures and subsequent significant developmental delay. Here, we identified an individual (ID139) who carried a missense variant c.1387A > G (p.Asp813Glu) in *CDKL5* on the X chromosome. This female was referred at the age of 3 years. Her developmental history indicates the manifestation of arrested development at the age of 1 year. At 3 years of age, the family reported sleeping difficulties; at 8 years old, the family reported obesity, a lack of motor coordination, and profound hyperactivity. The variant in *CDKL5* was predicted to be deleterious using SIFT and MutationTaster and was present in the ExAC database at a rate of 2 × 10^−5^. This variant appears to be a plausible cause of autism and developmental regression in ID139.

Genetic variants in *GRIN2A* are a major cause of Landau Kleffner syndrome. Patients ID090 and ID113 carried the same variant in *GRIN2A* (see above). In addition, ID031 carried a different variant, *GRIN2A* c.904G > T (p.Ala302Ser), which was absent from the ExAC database and present in the gnomAD database at a rate of 7.98 × 10^−6^. This variant was predicted to be deleterious. This boy was referred at the age of 3 years, 3 months after the onset of seizures and developmental arrest. His seizures were classified as atypical absences, myoclonic, axial tonic, and generalized tonic–clonic. The observation of three individuals with probable pathogenic variants in *GRIN2A* suggests that the dysfunction of *GRIN2A* may account for a substantial (2.2%) proportion of cases in this sample of individuals with regressive autism. 

Genetic variants in *SCN1A* have been associated with Dravet syndrome and other severe epileptic encephalopathies [24,36]. Patients with Dravet syndrome commonly present severe forms of epilepsy starting within the first year of life and the deterioration of intellectual development at around 2 years of age. The female ID037 and male ID121 carried novel splice-site and missense variants in *SCN1A*. Both individuals had developmental regression. The female’s seizures manifested at the age of 3 months as febrile seizures, which were followed by focal motor seizures in the right hand at 4.5 months and generalized tonic–clonic seizures at the age of 6 months; she was resistant to antiepileptic drugs. The male also manifested attention deficit, extreme hyperactivity, severe expressive speech delay with limited receptive language skills, stereotypic and repetitive movements, and cranial dysmorphology (brachycephaly). 

Mutations in *PCDH19* on the X chromosome cause epilepsy and intellectual disability preferentially in females, which is also known as Dravet-like syndrome [16]. Few males are affected, and it has been postulated that in hemizygous transmitting males, *PCDH11Y* on the Y chromosome likely compensates for the function of *PCDH19* [37]. We identified a female individual, ID051, who carried a missense variant c.121A > G (p.Asn41Asp) in *PCDH19*. This variant is absent in the ExAC database and present in the gnomAD database at a rate of 6.03 × 10^−6^. The patient manifested focal epilepsy with pseudoabsences, macrocephaly, cranial dysmorphology, decreased muscular tone, and macrosomia. Her developmental history indicates the onset of regression at the age of 16 months, first in the cognitive and then in the motor domains. Due to phenotypic presentation with macrosomia, Sotos Syndrome was considered in the differential diagnosis, but genetic testing did not detect any aberrations in the *NSD1* gene. Identification of the variant in *PCDH19* provides a possible molecular diagnosis to this patient and suggests the disturbance of shared pathways in regressive autism and Dravet-like syndrome.

The malfunction of *UBE3A* on the maternal allele is known to cause Angelman syndrome, which is characterized by intellectual disability, ataxia, severe speech impairment, and other neurological and behavioral features. Psychomotor regression is rarely seen or reported among individuals with Angelman syndrome, but there was some evidence for a linkage of psychomotor regression with *UBE3A* and its adjacent regions on chromosome 15 [17]. We identified an individual (ID029) who carries a novel missense variant c.2439C > G (p.Asp813Glu) in *UBE3A*. This variant was absent from the ExAC and gnomAD databases and was predicted to be damaging using SIFT and PolyPhen. The girl was referred at the age of 4 years with absence epilepsy and developmental arrest, intellectual disability, severe speech impairment, and problems with active movements (quadriparesis) and balance (ataxia). The history indicated a delay in motor and intellectual development but with progress in development until the age of 18 months, when she started to fall down frequently due to clumsiness and developed a spastic gait. Specific facial features and excitability were interpreted as suggestive of Angelman Syndrome. An allele-specific DNA methylation analysis of the promoter region of the *SNRPN* gene (chromosome 15q11.2) was performed and the outcome was normal. 

Christianson syndrome is caused by genetic variants in *SLC9A6* on the X chromosome. About 50% of patients with Christianson syndrome experience developmental regression at some point and commonly have intellectual disability, microcephaly, absent speech, ataxia, and seizures [2]. We identified one female (ID030) who carried a missense variant c.171C > G (p.Ile57Met) in *SLC9A6*. At the age of 11 months, she developed flexor infantile spasms and later developed generalized tonic–clonic seizures. From 2 years of age, developmental regression was noted; in addition, spastic quadriparesis and ataxia developed. Evidence for the pathogenicity of this variant has been conflicting. The variant is present in the ExAC database at a rate of 4.12 × 10^−5^ and was predicted to be damaging using SIFT and MutationTaster. It was classified in one other case with Christianson syndrome as likely benign and in another case with unspecified conditions as of uncertain significance (ClinVar# 379120). We would like to bring attention to this variant, as we observed a second case of an individual with a severe neurological phenotype and the presence of this rare genetic variant in *SLC9A6.*

### 3.4. Individuals Carrying Multiple Variants with Evidence Supportive of Pathogenicity

We also identified two individuals who carried variants with evidence supportive of pathogenicity in two different genes. A male (ID104) was found to have a heterozygous missense variant c.989A > T (p.Lys330Met) in *EHMT1* and a missense variant c.1777C > G (p.Leu593Val) in *SLC9A6*. Limited clinical information was available about this male. The variant in *EHMT1* was absent from the ExAC and gnomAD databases. De novo variants in *EHMT1* have been reported in individuals with autism, but developmental regression has not been reported [24]. Using SIFT and PolyPhen, the c.1777C > G variant in *SLC9A6* was predicted to be damaging, but a different variant at the same amino acid, c.1777C > T (p.Leu593Phe), was found in the ExAC database at a rate of 8.24 × 10^−6^.

A male (ID041), unrelated to ID104, carried heterozygous missense variants c.1513G > A (p.Gly505Ser) in *EHMT1* and c.353A > G (p.Asn118Ser) in *MFSD8*. He was seen at 7 years and 10 months and, at that time, was severely developmentally delayed in multiple domains (motor, cognitive, and language). Using Polyphen and MutationTaster, variants were predicted to possibly be damaging, but they were present in the ExAC database (*EHMT1* c.1513G > A at a rate of 4.95 × 10^−5^, *MFSD8* c. 353A > G at a rate of 8.24 × 10^−6^). As stated above, a heterozygous variant in *EHMT1* may not result in developmental regression. On the other hand, variants in *MFSD8* have been reported to cause autism and developmental regression, but only in an autosomal recessive condition [5]. *EHMT1* encodes a methyltransferase that specifically methylates histone H3K9me1 and H3K9me2 in euchromatin. *MFSD8* likely encodes a protein that transports molecules across the lysosomal membrane, with unknown specificity. This case suggests that ASD with regression may stem from a combinatorial effect of a partial loss of functions in both pathways.

## 4. Discussion

Autism spectrum disorder is a genetically heterogeneous and common developmental disorder that affects communication and behavior, with approximately one-third of individuals manifesting a regression of acquired skills. Various factors that may cause developmental regression include genetic predispositions; alteration in mechanisms critical at certain developmental stages, such as synaptic pruning [38]; infections or environmental triggers; and a combinatorial effect of genetic predisposition and environmental triggers. 

Given a lack of appreciation for the genetic landscape of regressive autism, we conducted a complete literature search for genes that were reported in the comorbidity of developmental regression with or without autism prior to August 2015, which formed a list of 47 genes. Using a preselected cohort of 134 individuals who had a diagnosis of autism and developmental regression, we assessed the likelihood of identifying variants in these 47 genes. It should be noted that even in this fairly homogeneous subtype of autism, clinical heterogeneity was substantial. Co-occurrence between seizures and developmental regression has been well-established [39], which was also observed in our cohort. In this study, we regard seizures, developmental regression, intellectual disability, autism, and other clinical manifestations all as traits under the spectrum of neurological disorders. Overall, we found variants with evidence supportive of pathogenicity in 15 individuals.

Interestingly, from our cohort with specific clinical manifestations, which was not large in size, we identified two recurrent variants, *GRIN2A* c.28C > A and *PLXNB2* c.742C > T, which are rare variants in the general population. This finding supports the role of *GRIN2A* and *PLXNB2* in the molecular etiology of autism and developmental regression. We also identified variants in genes that are linked to neurological syndromes, such as *MECP2, CDKL5, GRIN2A, SCN1A, PCDH19, UBE3A,* and *SLC9A6*. Developmental regression is frequently seen in patients with Rett Syndrome, Landau Kleffner Syndrome, and Christianson Syndrome. However, it has rarely been reported in other neurological syndromes, such as Dravet or Dravet-like syndrome and Angelman syndrome. Our findings provide further evidence that genetic risk factors of these syndromes also predispose one to developmental regression and autism. 

Some individuals were found to carry variants in multiple genes, which is in accordance with the idea of oligogenic heterozygosity in the molecular etiology of neurological disorders [40]. Genes that contributed in such a combinatorial manner in our study include *SCL9A6*, *EHMT1*, and *MFSD8*, which are involved in a variety of biological processes, such as cellular homeostasis, histone modification, and amino acid metabolism. Investigations into how oligogenic events increase one’s risk for ASD and regression will unravel new disease mechanisms.

The ACMG criteria we used in the study to define likely pathogenic variants was stringent, and we wondered whether there were variants in these 12 genes in the individuals without significant findings. Upon re-examination of those individuals, we found an additional 29 non-intronic and non-synonymous variants absent in the ExAC database in 15 individuals. The predicted consequence of these variants included the 5′ UTR variant (1 variant), splice region variant (5 variants), frameshift (7 variants), in-frame deletion or insertion (2 variants), missense (13 variants), and stop-gain (1 variant). Given the genetic heterogeneity underlying ASD and developmental regression, these variants could contribute to genetic predisposition in the disease conditions. 

The candidate approach that we used to explore the genetic risk factors in autism and developmental regression allowed rapid screening for variants throughout the samples, and the subsequent variant analysis process was relatively straightforward. However, this study has several limitations: (1) the candidate gene approach limits discovery to genes already described in their respective phenotypic contexts; (2) the lack of parental samples prevented us from determining inheritance vs. de novo status; (3) our study had a relatively small sample size. Future studies are needed to further investigate the genetic landscape of autism with regression, and genome-wide studies will be required to identify novel disease genes for this disease mechanism.

## 5. Conclusions

Our study provides additional insights into the relationship between autism and developmental regression by interrogating a set of genes that are associated with this comorbidity. The application of next generation sequencing in a sample of 134 children with autism and developmental regression highlights the roles of single gene variants in *MECP2, CDKL5, GRIN2A, SCN1A, PCDH19, UBE3A,* and *SLC9A6*, as well as oligogenic events (variants in *EHMT1* and *SCL9A6, EHMT1* and *MFSD8*) that may disrupt multiple biological processes.

## Figures and Tables

**Table 1:** Gene list for variant screening.

	Mode of Inheritance
*ATXN2*	x-linked
*EHMT1*	x-linked
*CDKL5*	x-linked
*PCDH19*	x-linked
*TMLHE*	x-linked
*MECP2*	x-linked
*SLC9A6*	x-linked
*ATP13A4*	Autosomal dominant
*ATP13A5*	Autosomal dominant
*CDH13*	Autosomal dominant
*CDH4*	Autosomal dominant
*CDH9*	Autosomal dominant
*GRIN2A*	Autosomal dominant
*HSBP1*	Autosomal dominant
*LRFN5*	Autosomal dominant
*MACROD2*	Autosomal dominant
*MBD5*	Autosomal dominant
*MDGA2*	Autosomal dominant
*RELN*	Autosomal dominant
*SCN1A*	Autosomal dominant
*SGSH*	Autosomal dominant
*SHANK3*	Autosomal dominant
*STXBP1*	Autosomal dominant
*TCF4*	Autosomal dominant
*UBE3A*	Autosomal dominant
*ADSL*	Autosomal recessive
*ALDH5A1*	Autosomal recessive
*FOXG1*	Autosomal recessive
*MFSD8*	Autosomal recessive
*PLCB1*	Autosomal recessive
*PRSS12*	Autosomal recessive
*SCN8A*	Autosomal recessive
*MTHFR*	Autosomal recessive
*DIAPH3*	Autosomal recessive
*FGF12*	Involved in copy number variations
*HRASLS*	Involved in copy number variations
*OPA1*	Involved in copy number variations
*SCN2A*	Involved in copy number variations
*ATXN7*	Involved in copy number variations
*CDH6*	Involved in copy number variations
*CNTNAP2*	Involved in copy number variations
*CTNNA3*	Involved in copy number variations
*GRIN2B*	Involved in copy number variations
*HDAC10*	Involved in copy number variations
*KCNQ2*	Involved in copy number variations
*KIF26B*	Involved in copy number variations
*PLXNB2*	Involved in copy number variations

**Table 2:** Summary of the clinical and genetic data of individuals with pathogenic variants.

Indivi-Dual ID	Sex	Onset Age of Regression	Variants	ACMG Classify-Cation	Seizures	Language Impair-Ment	Motor Impair-Ment	Additional Notes
090	M	36 months	*GRIN2A* c.28C > A p.Leu10Met	PM2	No	Yes	No	
113	M	36 months	*GRIN2A* c.28C > A p.Leu10Met	PM2	No	Yes	Yes	Stereotypies
031	M	39 months	*GRIN2A* c.904G > T p.Ala302Ser	PM2	Yes	Yes	No	
086	M	36 months	*PLXNB2* c.742C > T p.Arg248Cys	PP3	Yes	Yes	Yes	
110	F	14 months	*PLXNB2* c.742C > T p.Arg248Cys	PP3	Yes	Yes	Yes	Stereotypies, hand tremor
037	F	6 months	*SCN1A* c.4852 + 1G > T	PM2	Yes	Yes	Yes	Convulsions
121	M	24 months	*SCN1A* c.3269G > C p.Ser1090Thr	PM2	Yes	Yes	Yes	Stereotypies, attention deficit
051	F	16 months	*PCDH19* c.121A > G p.Asn41Asp	PM2	Yes	Yes	Yes	Facial dysmorphology
073	F	6 months	*MECP2* c.439A > G p.Lys147Glu	PS1	Yes	Yes	Yes	Loss of vision, hand tremor
139	F	12 months	*CDKL5* c.1387A > G p.Lys463Glu	PP3	No	Yes	Yes	Sleep disturbances, obesity, severe temper tantrums, stereotypies, aggression
029	F	18 months	*UBE3A* c.2439C > G p.Asp813Glu	PM2	Yes	Yes	Yes	Ataxia
030	F	Not available	*SLC9A6* c.171C > G p.Ile57Met	PP3	Yes	Yes	Yes	Spastic quadriparesis and ataxia, sleep disturbances
104	M	Not available	*EHMT1* c.989A > T p.Lys330Met & *SLC9A6* c.1777C > G p.Leu593Val	PM2 (for EHMT1) & PP3 (for SLC9A6)	Not available	Not available	Not available	
041	M	12 months	*EHMT1* c.1513G > A p.Gly505Ser & *MFSD8* c.353A > G p.Asn118Ser	PP3 for both	No	Yes	Yes	
137	M	11 months	*SCN2A* c.5549A > G p.Asp1850Gly & *MTHFR* c.136C > T p.Arg46Trp	PM2 for both	No	Yes	Yes	
